# Mental Health Services and Public Safety: Substance Abuse Outpatient Visits Were Associated with Reduced Crime Rates in a Swedish Cohort

**DOI:** 10.1371/journal.pone.0137780

**Published:** 2015-09-10

**Authors:** Natalie Durbeej, Tom Palmstierna, Ingvar Rosendahl, Anne H. Berman, Marianne Kristiansson, Clara Hellner Gumpert

**Affiliations:** 1 Department of Clinical Neuroscience, Center for Psychiatry Research, Stockholm County Council, Karolinska Institutet, Stockholm, Sweden; 2 Forensic department Brøset, Centre for Research and Education in Forensic Psychiatry, Division of Psychiatry, St. Olav's University Hospital, Trondheim, Norway; 3 Department of Neuroscience, Faculty of Medicine NTNU, Trondheim, Norway; 4 Stockholm Center for Dependency Disorders, Stockholm County Council, Stockholm, Sweden; Institute of Psychiatry, UNITED KINGDOM

## Abstract

Substance abuse is related to offending and substance abuse treatment has been associated with reductions in criminal behavior. This cohort study aimed to explore the relationship between participation in substance abuse interventions and general criminal recidivism among offenders with a combination of mental health problems and substance use problems. In total, 150 Swedish offenders with self-reported mental health and substance use problems were followed for approximately three years with regard to participation in substance abuse interventions and criminal recidivism. Participants with at least three planned visits to specialized outpatient substance abuse clinics had a substantially reduced risk of reoffending as compared to those with fewer than three such visits (HR = 0.47, 95% CI 0.29–0.77). For those with at least three planned visits, general criminal recidivism was reduced by 75% during periods of participation in outpatient visits, as compared to periods of non-participation (HR = 0.25, 95% CI 0.11–0.60). For offenders with mental health problems and substance use problems, outpatient substance abuse interventions could be regarded as important from a clinical risk management perspective, and be encouraged.

## Introduction

The associations between substance abuse, mental health problems, and offending are well documented in research. Comorbid substance abuse and mental health problems increase the risk of criminal behavior and need to be targeted not only to safeguard the health of the individual but also to reduce the risk of reoffending [1–3]. From this perspective, offenders with comorbid mental health and substance use problems constitute a population of relevance for public safety. After release from prison or forensic psychiatric treatment, many have complex treatment needs and substantial risk of criminal recidivism [4–5]. Research has emphasized the importance of providing treatment to these individuals [4–5] but few studies have explored their treatment participation and associated outcomes. A Swedish study concluded that participation in outpatient substance abuse treatment was associated with a reduced risk of reoffending in this population [6]. This finding was in line with international research showing the potential of substance abuse interventions in reducing criminal behavior among offenders [7–8].

In addition to substance abuse and mental health problems, several other risk factors of criminal behavior have been identified. Risk factors of future criminal behavior are often categorized as either static or dynamic, where the former are stable over time, and the latter are subject to change. Some of the static risk factors comprise of male gender, younger age, previous offending, and family criminality or violence [9–11], whereas some of the dynamic risk factors (commonly referred to as criminogenic needs) include interpersonal conflicts, antisocial personality and companions, impulsivity, and importantly, substance abuse [2,9,12]. Research suggests that dynamic risk factors perform as well as static risk factors in predicting future criminal behavior [10,13] and that dynamic risk factors are important targets for interventions aiming to reduce the risk of criminal recidivism [2,12].

A number of risk assessment tools comprising both static and dynamic risk factors have been developed in order to evaluate risk of future criminal behavior and extensive research has explored their predictive validity. As one example, higher violence risk according to the Historical, Clinical and Risk Management scale (HCR-20) have predicted criminal recidivism in various offender samples [14]. Included in the HCR-20 is psychopathy, another important risk factor of criminal behavior, particularly violence [15]. According to the Canadian psychologist Robert Hare, the construct of psychopathy includes personality traits describing a deceitful and deficient affective style, as well as impulsive and antisocial features [16]. Previous studies have confirmed that psychopathy is prevalent and predictive of criminal recidivism among offenders, including those with mental health problems, and that its predictive capacity is driven by the antisocial features [15–17].

To conclude, offenders with mental health problems and substance use problems may provide challenges to clinical care and have high risk of crime relapse. The need to refine and elaborate treatment strategies for this population is considered highly important [3,5]. Substance abuse may be targeted with the aim to reduce criminal behavior given that substance abuse interventions are widely available [2]. Although studies have shown that participation in substance abuse interventions is associated with reductions in crime [7,8], little research has explored whether substance abuse intervention participation may be related to lower crime rates among offenders with combined mental health and substance use problems [6,18,19]. Studies on this topic have mainly explored the effect of correctional interventions and used rather short follow-up periods (i.e. 12 months). Thus, research on the efficiency of community interventions for this population is rather scarce [18]. When released from prison or compulsory inpatient forensic psychiatric care into the community, offenders with mental health problems and substance use problems face many difficulties and are likely to have remaining problems [3,5]. Community interventions provide a realistic context in which it is possible to practice new skills, and have been highlighted as important for this, as well as other offender populations, in order to reduce problems and crime relapse rates [3,20,21]. Exploring the participation in community interventions and crime outcomes among offenders with combined mental health and substance use problems could add useful knowledge in order to facilitate treatment provision for this population. Treatment may be associated with reductions in criminal behavior which in turn may lead to an increased well-being and safety for citizens in society.

### Aims of the current study

We aimed to explore the relationship between participation in substance abuse interventions and general criminal recidivism among offenders with combined mental health and substance use problems. We also sought to control for other factors known to affect the risk of criminal recidivism and based on the results of an earlier retrospective study, we hypothesized that participation in planned substance abuse outpatient visits would be associated with a reduced risk of crime relapse [6].

## Materials and Methods

### Treatment context

In Sweden, two principal systems provide substance abuse interventions: the social services system managed by the local municipalities and the health care system managed by the County Councils. The former provides non-medical and non-psychiatric interventions such as counseling, or economic or housing assistance. The latter provides specialist medical and psychiatric treatment such as emergency services and/or pharmacological treatment. In Stockholm County, all such interventions are provided through two separate Centers of Dependency disorders offering treatment in both inpatient and outpatient settings. In Sweden, all interventions provided by the social services and the health care systems are easily available at no or low cost to the individual.

In the current study, we sought to explore the effect of two substance abuse interventions on general criminal recidivism: a) participation in planned visits to specialized substance abuse outpatient clinics provided by the health care system and b) residence in housing that required abstinence monitoring (“dry housing”) provided by the social services system. A planned visit involved any planned appointment with a service provider (e.g. a psychologist, psychiatrist, counselor, or nurse) at a medical outpatient clinic specialized on dependency disorders in Stockholm County. Such a visit generally includes breath and blood analyses for alcohol consumption, urine analyses for illegal drug use, counseling and, when required, pharmacological treatment. Dry housing included residence at a supportive and/or treatment facility, where abstinence monitoring was mandatory. The rationale for selection of these interventions was to explore the impact of interventions that included monitoring of abstinence. Both interventions were non-standardized, allowing for differences between visits and between residences.

### Participants

The present study was part of a longitudinal Swedish cohort study of offenders with comorbid mental health and substance use problems (“Mental disorder, Substance Abuse, and Crime” (MSAC)) [22]. We recruited participants among suspected offenders referred for forensic psychiatric assessment (FPA) during the remand period. According to the Swedish penal system, a person who has committed a crime under the influence of a severe mental disorder (a legal term that includes conditions such as psychotic states, severe depression and/or personality disorders with compulsive behaviour or uncontrollable impulsivity) should not be sentenced to prison, but instead referred for compulsory forensic psychiatric treatment. Thus, a suspect who displays symptoms of a serious mental health condition is referred for FPA. Although less than half of those referred are judged to have a severe mental disorder, a majority receive one or more psychiatric diagnoses.

Inclusion criteria for participation in the MSAC study were a) referral for FPA, b) registered residence in Stockholm County and c) hazardous use of alcohol and/or illicit drugs Our aim was to include a group of individuals with multiple problems, that is, with mental health and substance use problems, manifest criminal behavior, and risk of reoffending. Instead of using diagnostic procedures, we used assessment tools in order to measure symptom levels of these problems. The rationale for this decision was the particular challenges this group constitutes to community treatment services as well as to the prison and probation services, regardless of diagnosis. We referred to the participants as having mental health problems instead of mental disorders, given that mental disorders have been considered to lie on a continuum with overlapping psychiatric symptoms, rather than being separate entities [23].

Recruitment to the study took place between February 2, 2006 and April 21, 2009. In total, 252 individuals fulfilled the above inclusion criteria and were invited to participate in the study. Of those invited, 207 (82%) gave their written consent to study participation and were subject to a baseline interview and follow-ups on three occasions: 1) shortly before release from prison or forensic psychiatric hospital or six months after study inclusion (for those sentenced to probation), 2) six months after the first follow-up, and, 3) 12–18 months after the second follow-up. Baseline interviews and follow-ups took place between February, 2, 2006 and January, 18, 2012, a mean time period of 34.17 months (*SD =* 9.19 months, range = 19–63 months). The mean time between baseline and the first follow-up assessment for participants released from prison or forensic psychiatric hospital was 14.98 months (*SD* = 9.85 months, range = 1–43 months).

Among the 207 MSAC participants, 39 individuals were still in prison or inpatient forensic psychiatric care during the entire study period and were thus not available for follow-up. Also, one died before the first follow-up, two could not be found and seven declined further study participation after the baseline interview. Altogether, 158 individuals were subject to the first follow-up. Among these, we excluded another four who were found not guilty of the index crime, three who declined further study participation and one who was sentenced for the index crime after the study endpoint (31 October 2011). Thus, the final sample comprised 150 individuals who were followed with regard to participation in substance abuse interventions and general criminal recidivism.

### Data sources

Screening of participants for hazardous use of alcohol and/or illicit drugs was performed with the Alcohol Use Disorders Identification Test (the AUDIT), a ten item questionnaire (total score range: 0–40) developed by the World Health Organization (WHO) and the Drug Use Disorders Identification Test (the DUDIT), an eleven item questionnaire (total score range: 0–44) developed at Karolinska Institutet [24,25]. The items of the AUDIT and the DUDIT concern use of alcohol and illicit drugs respectively (e.g. frequency and quantity), symptoms of dependency (e.g. impaired control over use and increased salience of use), and harmful use (e.g. blackouts and injuries). Both questionnaires have demonstrated adequate validity and reliability in various populations and settings [26,27], also among offenders with mental health problems and substance use problems [28]. Hazardous use of alcohol was defined as an AUDIT score of 8 or more points for men and 6 or more points for women [24]. The rationales for the lower scores for females are that women tend to develop a higher blood-alcohol-concentration level after the same amount of alcohol intake relative to men, and that the risk for medical alcohol-related consequences is higher for women than for men [29]. Given that all drug use in Sweden is illegal, hazardous use of illicit drugs was defined as a DUDIT score of 1 point or more for both men and women [30].

The participants were assessed with the Addiction Severity Index, sixth version (ASI-6), [31,32] an interview covering problem severity in nine domains: medical, employment, alcohol, drug, legal, psychiatric, family/social problems, family/social support, and child problems. Also, information was collected from the Central Archive of The National Board of Forensic Medicine. This register contains information on all individuals subject to FPA in Sweden since 1934 and includes data such as actual and previous criminality, sentences, social situation, mental health status, and demographic data. The register has been stored in a computerized database with complete coverage since 1987. Data on index crimes and associated sentences for the study participants were collected from this database.

Psychopathic personality traits were assessed with the Psychopathy Checklist-Revised (PCL-R) [16], a tool comprising 20 items. The items of the PCL-R are rated on a 3-point scale (0,1 or 2) to indicate absence, partial presence, or presence of the trait or behavior referenced in the item (total score range: 0–40). In Sweden, a cut-off score of 26 points for psychopathy has been proposed among offenders with mental health problems [33].

The PCL-R items can be divided into four factors. The interpersonal facet (items: glibness/superficial charm, grandiosity, pathological lying, and manipulation for personal gain, total score range: 0–8), the Affective facet (items: lack of remorse/guilt, shallow affect, lack of empathy and failure to accept responsibility, total score range: 0–8), the Lifestyle facet (items: proneness to boredom, parasitic lifestyle, lack of realistic long-term goals, impulsivity, irresponsibility, total score range: 0–10) and the Antisocial facet (items: poor behavioral control, early behavioral problems, juvenile delinquency, criminal versatility, revocation of conditional release, total score range: 0–10). Two of the items, sexual promiscuity and many short-term marital relationships do not load on any of the facets. The total PCL-R as well as the four-factor model has demonstrated adequate validity and reliability in various offender samples [34,35].

Risk for future violence was assessed with the Historical, Clinical, and Risk-Management scale (HCR-20) [14], a tool with 20 items referring to static and dynamic risk factors of future violence. As with the PCL-R, the HCR-20 items are rated on a 3-point scale to indicate absence, partial presence, or presence of the risk factor referenced in the item (total score range: 0–40). The HCR-20 has proven to have adequate validity and reliability both in international and Swedish research on offenders [36–38].

Data on participation in planned visits to substance abuse outpatient clinics were extracted from the official registry on health care utilization in Stockholm County Council. This registry has almost complete coverage of the number of outpatient visits to both public and private service providers of substance abuse interventions in the county [39]. Data on dry housing residence were provided by the social services system in Stockholm County, gathered through social services records requested from the local districts in the municipalities.

Data on general criminal recidivism, defined as new convictions after inclusion to the study/baseline assessment, were collected from the registry of the National Council for Crime Prevention, which comprises data for all convictions in Sweden since 1973. In order not to follow up criminality of deceased or emigrated individuals, information was also gathered from the Swedish Tax Authority, covering information on all citizens residing in Sweden.

### Procedure

Five research assistants performed the assessments of the study [22,28]. Recruitment and baseline assessments took place at the National Board of Forensic Medicine in Huddinge (agency responsible for performing FPAs), and at the Huddinge and Kronoberg remand prisons; all located in Stockholm. All individuals who fulfilled the inclusion criteria (see above) of the MSAC-study were offered study participation. For follow-up purposes, the participants were contacted individually by telephone. The follow-up assessments took place at libraries or cafés in the Stockholm city center, or in remand or prison settings. All assessments of the study were conducted face-to face (baseline and follow-ups).

The research assistants had either clinical experience of compulsory inpatient forensic psychiatric treatment in FPA units or at least a B.Sc degree in behavioral sciences. All were formally trained and authorized to use the assessment tools (i.e. the ASI-6, the PCL-R and the HCR-20). The training involved lectures on the use of the instruments in research settings, interviewing style, rules for coding, case studies, role play, and lasted at least two days.

All raw-data were stored securely in a fire-proof cabinet at the National Board of Forensic Medicine. When participants were assessed elsewhere (e.g. at follow-ups), the raw-data was securely handled and immediately transported to the National Board of Forensic Medicine for storage after the assessment was completed. The data were also stored electronically. To protect the integrity of the participants, all personal identification numbers and names were removed from the data files.

### Variables and statistical analyses

The main independent variables of the study were two variables for participation in substance abuse interventions. In the statistical analyses (see below), they served as time dependent covariates (i.e. as variables changing over time) and were treated as dichotomous yes/no variables defined as a) at least three planned visits to a specialized substance abuse outpatient clinic during the follow-up period and b) residence in dry housing at least once during the follow-up period.

The number of planned outpatient visits was based on the assumption that at least three planned visits could indicate some stability in treatment attendance and therefore be considered as intervention participation [28]. We performed a number of bivariate sub analyses in order to explore the relationship between at least one, two, or three planned visits, respectively, and general criminal recidivism. Although all three categories were significantly associated with lower crime relapse rates, two planned visits and three planned visits yielded higher and similar impact on the outcome, relative to one planned visit. Given the little difference in impact between at least two and three planned visits, and our assumption that two visits would not be sufficient to indicate stability in treatment attendance, we used at least three planned outpatient visits as a definition of intervention participation.

We also included a number of static independent variables, known to affect the risk of reoffending. These were age, the number of convictions prior to the index crime, the four PCL-R facets and the HCR-20 violence risk total score. A previous study on the current population identified subgroups with unique problem profiles [4]. The subgroups were defined based on a cluster analysis of the ASI-6 problem severity domains and with reference to the term “triply troubled”, referring to individuals with a combination of mental health problems, substance use problems, and criminal behavior [40]. The identified subgroups included 1) “Less troubled”, individuals with low degrees of various problems, 2) “Severely triply troubled”, individuals with severe legal-, psychiatric-, alcohol-, drug- and family/social problems, 3) “Triply troubled with medical problems”, individuals with elevated legal-, medical-, psychiatric and drug problems, and 4) “Working triply troubled”, individuals with low levels of employment problems and medium levels of alcohol-, psychiatric-, and legal problems [4]. Dichotomous yes/no variables for membership in any of the above subgroups were also included in the analyses.

The dependent variable of the study was general criminal recidivism, which also was treated as a dichotomous yes/no variable, defined as relapse into any crime (including assault, narcotic-related crimes, theft, traffic-related crimes, robbery, sexual crimes and other crimes like fraud or vandalism) during the follow-up period.

Means, standard deviations, frequencies, and ranges were used to describe the participants. In addition, one-way analysis of variance (ANOVA) and Tukey post-hoc test were used to compare means, and chi-square analyses were used to test differences in prevalence. The following survival analyses were used to explore the relationship between participation and general criminal recidivism: an extended Cox-proportional-hazards regression model with survival curves [41] and a stratified Cox-proportional-hazards regression model (see below) [42]. Before performing these analyses, we calculated time at risk, defined as time spent living in the community, outside prison or inpatient forensic psychiatric treatment. For participants sentenced to probation in association with the index crime, time at risk started immediately at the date of the sentence of the crime. For those sentenced to prison or inpatient forensic psychiatric treatment, time at risk began at the date of release from prison or forensic psychiatric hospital, respectively. If the participant was reconvicted, time periods in prison or hospital during the follow-up period were subtracted from time at risk.

We sought to explore time to multiple reconvictions. If a reconviction occurred, the individual was followed until the date of the main crime of the conviction. At that date, time at risk was reset at 0 and the participant was followed until another conviction or the study endpoint (31 October 2011). If no conviction occurred before the study endpoint, time at risk was right-truncated by that date. Among the 150 participants, three emigrated and five died before the study endpoint but were followed until the date of death or emigration. All emigrations occurred between the second and the third follow-up assessment of the MSAC-study. The deaths occurred between the first and second follow-ups (two cases), the second and third follow-ups (1 case) as well as the third follow-up and the study endpoint (two cases). The mean length of time at risk for all participants was 33.31 months (range = 1.40–63.26 months).

To explore the relationship between participation in substance abuse interventions and general criminal recidivism, an extended Cox-proportional-hazards regression model was estimated [41]. This model can be regarded as an extended version of the original Cox model, allowing for analysis of both static independent variables and time-dependent covariates in relation to multiple events (e.g. reconvictions). The analysis was performed in two blocks. In the first block, only the static variables were entered, whereas in the second block, the time-dependent covariates (i.e. the variables for participation in substance abuse interventions) were added to the model. This procedure was performed in order to evaluate whether the model would improve when the substance abuse intervention participation variables were added as predictors of reoffending. Given that we had no specific hypotheses about the order or importance of the independent variables in relation to the dependent variable, all independent variables were entered simultaneously in each block.

In order to visually describe the results from the model, and to explore the probability of committing a new crime during the follow-up period, survival curves from the extended Cox-proportional-hazards regression model were calculated. In this analysis, estimates for participants with a record of at least three planned visits to an outpatient clinic, and participants with fewer than three such visits during the follow-up period were explored. In addition, the estimates were tested with a log-rank test.

Finally, to explore the risk of general criminal recidivism during periods of participation in substance abuse interventions compared with periods of non-participation in such interventions, we computed a stratified Cox-proportional-hazards regression model [42]. In this analysis, each participant served as his or her own control which adjusted for confounders that were constant within each participant during the follow-up period. Only the two substance abuse interventions participation variables were entered as predictors in this model. All analyses of the study were computed using SPSS, version 22.0 [43], and Stata, version 13 [44].

### Ethics statements

All participants were given oral and written information about study participation and signed a written consent form at recruitment for the MSAC-study. Ethical approval of the study was granted by the Regional Ethical Review Board in Stockholm, Sweden, on December 7, 2005 (2005/5:11).

## Results

### Sample characteristics

The sample comprised 136 (90.7%) men and 14 (9.3%) women with a mean age of 33.67 years (*SD* = 11.41 years, range = 17–61 years), multiple previous convictions and several years of regular alcohol and/or drug use (Table 1).

**Table 1:** Participant characteristics (*n* = 150)^a^.

Variables	*M* (*SD*)^b^	*Range*
Prior criminality and substance use^c^		
Mean no. of convictions prior to index crime	5.45 (9.34)	0–63
Mean no. of years with regular^d^ use of alcohol	6.53 (8.55)	0–40
Mean no. of years with regular^d^ use of illicit drugs	7.88 (9.73)	0–40
Prior psychiatric symptoms	*n* (%)	
Felt depressed or down most of the day	125 (83.3)	
Felt anxious, nervous or worried most part of the day	123 (82.0)	
Had trouble thinking/concentrating/remembering	122 (81.3)	
Pushed, hit, thrown things at, or used a weapon	113 (75.3)	
Had serious thoughts of committing suicide	99 (66.0)	
Had difficulty controlling temper/urges to hit or harm	99 (61.3)	
Had hallucinations	85 (56.7)	
Attempted suicide	79 (52.7)	
Any of the above psychiatric symptoms	147 (98.0)	
Prior treatment experiences	*n* (%)	
Treated for alcohol or drug use	95 (63.3)	
Had contact with outpatient psychiatric settings	91 (60.7)	
Index crimes^e^	*n* (%)	
Violent crimes^f^	113 (75.4)	
Narcotic-related crimes	11 (7.3)	
Sexual crimes	11 (7.3)	
Arson	8 (5.3)	
Property crimes	7 (4.7)	
Sentences associated with the index crime	*n* (%)	
Imprisonment	92 (61.3)	
Probation or fines	34 (22.7)	
Compulsory inpatient forensic psychiatric treatment	24 (16.0)	
PCL-R and HCR-20 scores	*M* (*SD*)	*Range*
Total PCL-R	14.23 (7.88)	1–33
Interpersonal PCL-R facet	1.57 (1.55)	0–6
Affective PCL-R facet	2.88 (1.95)	0–7
Lifestyle PCL-R facet	4.85 (2.73)	0–10
Antisocial PCL-R facet	3.28 (2.56)	0–10
Total HCR-20	16.52 (7.78)	1–31
Subgroup membership^g^	*n* (%)	
Less troubled^h^	26 (17.3)	
Severely triply troubled^i^	24 (16.0)	
Triply troubled with medical problems^j^	38 (25.3)	
Working triply troubled^k^	60 (40.0)	
Substance abuse intervention variables	*n* (%)	
≥ 1 planned visit to an outpatient clinic	75 (50.0)	
≥ 3 planned visits to an outpatient clinic	47 (31.3)	
Residence in dry housing	35 (23.3)	
	*M* (*SD*)	*Range*
Mean no of planned visits to an outpatient clinic	4.22 (8.05)	0–48
Mean no of weeks spent in dry housing on one occasion	9.73 (8.78)	1–31
Mean no of planned visits to an outpatient clinic (*n* = 75)^l^	8.44 (9.71)	1–48
Mean no of planned visits to an outpatient clinic (*n* = 47)^m^	11.87 (10.83)	3–48
General criminal recidivism		
Re-offended with at least one criminal conviction	76 (50.7)	

^a^ Data presented according to the sixth version of the ASI-6, the Central Archive of The National Board of Forensic Medicine, the PCL-R, the HCR-20, the official registry on health care utilization in Stockholm County Council, social services records, and the registry of the National Council for Crime Prevention.

^b^ *M (SD)* = Mean (Standard Deviation).

^c^ Data on prior criminality, substance use, psychiatric symptoms and treatment experiences concern the time period after 18 years of age.

^d^ More than three days per week.

^e^ Main crime at the index conviction.

^f^ Assault, murder/manslaughter, threat and robbery.

^g^ The subgroups were defined with reference to the term “triply troubled”, referring to individuals with a combination of mental health problems, substance use problems, and criminal behavior [4,40]. Two of the participants had not been assigned a subgroup membership.

^h^ Subgroup with low degrees of various problems.

^i^ Subgroup with severe legal-, psychiatric-, alcohol-, drug- and family/social problems.

^j^ Subgroup with elevated legal-, medical-, psychiatric and drug problems.

^k^ Subgroup with low levels of employment problems and medium levels of alcohol-, psychiatric-, and legal problems.

^l^ Among participants with at least one planned visit to an outpatient clinic.

^m^ Among participants with at least three planned visits to an outpatient clinic.

Self-reports of psychiatric symptoms were common, also including more severe symptoms such as hallucinations (56.7%). Over half had previously participated in treatment interventions targeting alcohol or drug use (63.3%), and/or had had contact with outpatient psychiatric settings (60.7%). During the follow-up period, 47 individuals (31.3%) had a record of at least three planned visits to an outpatient clinic, and 35 (23.3%) had resided in dry housing at least once. Among those with a record of at least three planned visits to an outpatient clinic (*n* = 47), 17 individuals (36.2%) had also lived in dry housing whereas 30 individuals had no such experiences. Among those with less than three planned visits (*n* = 103), 18 had lived in dry housing. A larger proportion of those with a record of at least three planned visits to an outpatient clinic had also lived in dry housing, relative to those with less than three planned visits, illustrating an overlap between substance abuse outpatient participation and dry housing residence (*χ* ^*2*^(1, 150) = 6.31, *p* < .05). Approximately half (50.7%) had reoffended with at least one criminal conviction.

In order to assess potential differences between participants who had a record of at least three planned outpatient visits and participants who had lived in dry housing, a number of comparative analyses were performed. Given the overlap between substance abuse outpatient participation and dry housing residence, four participant groups were compared on relevant variables (e.g. criminal history, mental health problems, substance use problems, homelessness, PCL-R scores and HCR-20 score): 1) participants with at least three planned outpatient visits only (*n* = 30), 2) participants who had lived in dry housing only (*n* = 18), 3) participants who had received both interventions (*n* = 17), and 4) participants who had not lived in dry housing and/or had a record of fewer than three planned outpatient visits (*n* = 85). Participants who had lived in dry housing only had a higher mean score on the antisocial PCL-R facet, relative to participants with at least three planned outpatient visits only (*F* (3,146) = 2.95, *p* < .05). None of the remaining comparisons yielded any significant differences.

### Substance abuse intervention participation in relation to criminal recidivism

According to Block II of the extended Cox-proportional-hazards regression model (Table 2), higher scores of the Antisocial PCL-R facet (Hazards Ratio [HR] = 1.16, 95% CI 1.06–1.27) and membership in the subgroup “Triply troubled with medical problems” (HR = 2.00, 95% CI 1.36–2.95) were associated with an increased risk of general criminal recidivism.

**Table 2:** Prediction of general criminal recidivism estimated by extended Cox-proportional-hazards regression (*n* = 148)^a^.

Block I	Standard Error	Hazard ratio (95% CI^b^)
Age	0.01	0.99 (0.97–1.00)
No. of convictions prior to the index crime	0.01	1.00 (0.97–1.02)
PCL-R variables		
Interpersonal PCL-R facet	0.08	1.12 (0.97–1.29)
Affective PCL-R facet	0.06	1.05 (0.94–1.18)
Lifestyle PCL-R facet	0.06	1.06 (0.94–1.19)
Antisocial PCL-R facet	0.05	1.19 (1.09–1.30)
Total HCR-20 score	0.02	0.97 (0.94–1.01)
Subgroup membership^c^		
Less troubled^d^	0.43	1.24 (0.63–2.11)
Severely triply troubled^e^	0.37	1.46 (0.90–2.40)
Triply troubled with medical problems^f^	0.35	1.90 (1.32–2.74)
Log pseudolikelihood –672.801		
Block II	Standard Error	Hazard Ratio (95% CI^b^)
Age	0.01	0.98 (0.96–1.00)
No. of convictions prior to index crime	0.01	1.00 (0.98–1.02)
PCL-R variables		
Interpersonal PCL-R facet	0.08	1.11 (0.96–1.29)
Affective PCL-R facet	0.06	1.07 (0.96–1.19)
Lifestyle PCL-R facet	0.06	1.05 (0.94–1.18)
Antisocial PCL-R facet	0.05	1.16 (1.06–1.27)
Total HCR-20 score	0.02	0.98 (0.94–1.01)
Subgroup membership^c^		
Less troubled^d^	0.40	1.22 (0.64–2.33)
Severely triply troubled^e^	0.37	1.43 (0.86–2.37)
Triply troubled with medical problems^f^	0.38	2.00 (1.36–2.95)
Substance abuse intervention variables		
≥ 3 planned visits to an outpatient clinic^g^	0.12	0.47 (0.29–0.77)
Residence in dry housing^h^	0.41	1.23 (0.64–2.37)
Log pseudolikelihood –668.320		

^a^ Two participants had not been assigned a subgroup membership and were therefore excluded from the analysis [4].

^b^ 95% CI = 95% Confidence Interval.

^c^ The subgroups were defined with reference to the term “triply troubled”, referring to individuals with a combination of mental health problems, substance use problems, and criminal behavior [4,40]. Membership of the subgroup “Working triply troubled” (i.e. the subgroup with low levels of employment problems and medium levels of alcohol-, psychiatric-, and legal problems) was used as a reference category. Each of the remaining subgroups was compared to this particular group (not shown in the table).

^d^ Subgroup with low degrees of various problems.

^e^ Subgroup with severe legal-, psychiatric-, alcohol-, drug- and family/social problems.

^f^ Subgroup with elevated legal-, medical-, psychiatric and drug problems.

^g^ Compared to <3 planned visits to an outpatient clinic.

^h^ Compared to no residence in dry housing.

**Fig 1:**
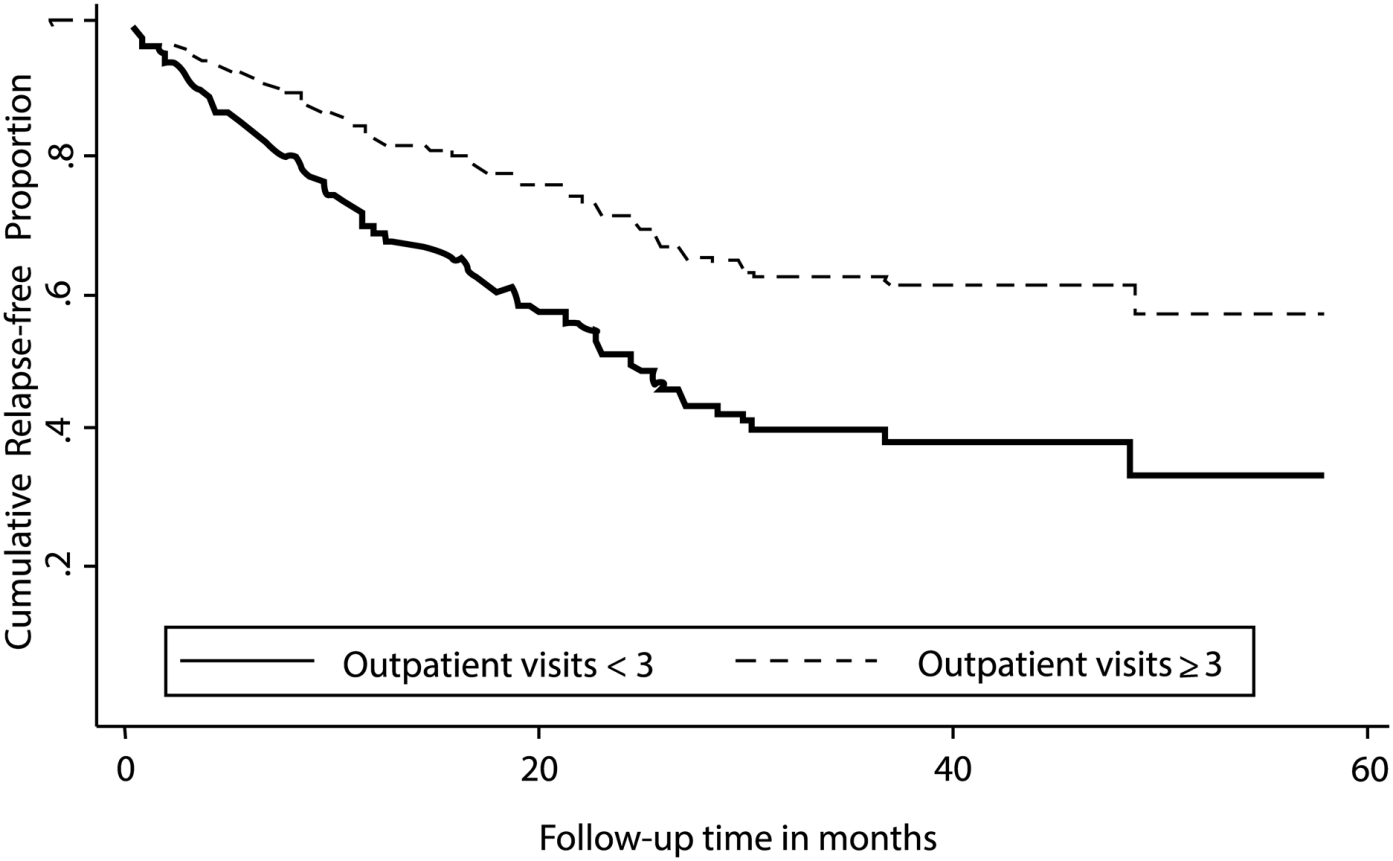
Survival curves from the extended Cox-proportional-hazards model for probability of criminal recidivism during follow-up. Estimates for participants with a record of at least three planned visits to an outpatient clinic and participants with fewer than three such visits. Log rank test: χ² = 8.87, *p* < .05 (*n* = 150).

Participants with at least three planned visits to an outpatient clinic had a reduced risk of reoffending relative to those with fewer than three such visits (HR = 0.47, 95% CI 0.29–0.77). However, dry housing residence as well as the remaining independent variables had no relation to the outcome. The prediction of general criminal recidivism was significantly improved when the substance abuse intervention participation variables were added as independent variables (χ^2^ = 4.48, *p* < .05). According to the survival curves from the extended Cox-proportional-hazards model (Fig 1), participants with at least three planned visits to an outpatient clinic had a lower probability of committing a new offense, relative to those with fewer than three such visits. The median survival time among the participants was 22.79 months (range = 2.09–24.42 months).

Finally, the stratified Cox-proportional-hazards regression model showed a 75% reduced rate of criminal recidivism (HR = 0.25, 95% CI 0.11–0.60) during outpatient unit contact periods as compared with periods of non-participation in planned outpatient visits, among participants with at least three planned outpatient visits. The association was not significant for dry housing residence (Table 3).

**Table 3:** Prediction of general criminal recidivism estimated by stratified Cox-proportional-hazards regression.

Substance abuse intervention variables	Number of participants	Standard Error	Number of convictions	Hazard Ratio (95% CI^a^)
Residence in dry housing^b^	35	0.31	7	0.50 (0.15–1.67)
≥ 3 planned visits to an outpatient clinic^c^	47	0.11	15^d^	0.25 (0.11–0.60)

^a^ 95% CI = 95% Confidence Interval.

^b^ Compared to no residence in dry housing.

^c^ Compared to <3 planned visits to an outpatient clinic.

^d^ Thirteen individuals had re-offended with 15 convictions.

## Discussion

The current study aimed to explore the relationship between participation in substance abuse interventions and general criminal recidivism among offenders with mental health and substance use problems. Our sample comprised individuals characterized by multiple previous convictions, mental health and substance use problems (i.e. with risk of re-offending). Many (50.7%) did relapse into crime, corroborating findings from earlier studies [5,6]. However, a striking observation was the relationship between participation in planned outpatient visits and lower crime relapse rates. This association was significant regardless of other factors known to be related to criminal recidivism (e.g. psychopathic personality traits) and was reinforced when we compared periods of participation and non-participation in such visits. Our findings are in line with earlier research showing an association between substance abuse interventions and lower crime relapse rates among offenders with multiple problems (i.e. criminal behavior, mental health problems and substance use problems) [6,18,19]. Also, our findings support previous studies highlighting the potential efficiency of outpatient interventions for this population [6,19].

Of the PCL-R facets, only the Antisocial PCL-R facet predicted general criminal recidivism, and this finding was concordant with previous research [17]. Also, membership in the subgroup “Triply troubled with medical problems” was associated with an increased risk of reoffending. Medical problems, along with psychiatric problems and substance use problems, have predicted criminal behavior in earlier research [45]. Thus, the higher risk of criminal recidivism among members of this subgroup might be explained by their severe problem profile, that is, a combination of drug-related-, psychiatric-, and medical problems. Taken together, individuals with such a broad problem severity and antisocial behavior seemed to be at particular risk of criminal recidivism. This stresses the importance of comprehensive assessment procedures and multimodal treatment packages in crime rehabilitation programs.

Our results suggested that participation in planned visits was related to substantially lower crime relapse rates, but that there was no relationship between dry housing residence and criminal recidivism. As described above, and in line with a previous study on the current study population [22], participants who had lived in dry housing only had a higher mean score on the antisocial PCL-R facet relative to participants with at least three planned outpatient visits only. This might imply a higher risk of criminal recidivism among the former participants in comparison with the latter. Hypothetically, rehabilitation of those living in dry housing could be challenging.

Furthermore, it is possible that the different nature of the two interventions could explain our results. A visit to a substance use outpatient clinic will primarily focus on the patient’s substance use problems, providing abstinence monitoring and access to specialized staff that can offer various interventions that may be individually tailored. Dry housing residence is an intervention primarily aimed at solving a social problem–housing—while at the same time requiring abstinence monitoring. Given this difference, a possible interpretation of our findings is that in a sample of offenders with multiple problems, abstinence monitoring only was not sufficient to impact the participants’ lives to the degree that the crime relapse rates were decreased. Such an interpretation would indicate that support and monitoring of former offenders with comorbid mental health and substance use problems require access to psychiatric care on the specialist level. The Swedish prison and probation service, forensic psychiatric services, as well as the community social services may need to consider the potentially crucial importance of access to specialist substance use medical competence.

It should be noted, however, that we did not have access to data on any details regarding what was provided within the framework of the interventions. Thus, future research should explore the efficiency of any specific elements of the two interventions (e.g. staff profile and competence). As far as we are aware, no previous study has explored the relationship between dry housing and criminal recidivism, but dry housing has been described as a suitable treatment alternative for individuals with comorbid mental health problems and substance use problems [46]. Thus, dry housing might have predicted such outcomes. The efficiency of dry housing in both Swedish and international contexts need to be further assessed before any firm conclusions regarding this intervention can be stated.

The finding that participation in planned visits was related to substantially lower crime relapse rates may interpreted as if offenders with mental health problems and substance use problems may benefit from some stability in their contact with the Swedish health care system. Given that previous treatment participation may facilitate future treatment participation [47], these individuals could also be invited and motivated to participate in substance interventions already in prison or during forensic psychiatric treatment. Given that substance abuse treatment programs are available in Sweden, allocation of community based, specialized outpatient substance abuse interventions could be regarded as an important aspect of a clinical risk management strategy. Participation and retention in these interventions could lead to an increased safety for citizens in society.

### Strengths and limitations

The conclusions that can be drawn from the results of the current study are limited due to the observational study design. Thus, the relationships between the independent variables and the outcome should be regarded as correlational rather than causal. During the entire follow-up period of the MSAC-study, 39 individuals were still in prison or inpatient forensic psychiatric care during the entire study period and were thus not available for follow-up. The fact that these individuals could not be included is a limitation, since it is likely that they had committed more serious types of index crimes, and displayed higher psychopathy scores, violence risk scores as well as higher crime relapse rates, relative to the participants available for the present study. Among the study participants, the total PCL-R and HCR-20 scores were about 14 and 16 points, which is not extremely high. Accordingly, the results should be inferred primarily to similar offenders; i.e. those with several convictions, mental health problems, substance use problems, some psychopathic personality traits and a medium risk of future violence. Given that all participants were registered in the Stockholm County, the results should foremost be generalizable to offenders in urban areas.

The substance abuse intervention participation variables did not concern participation in a defined substance abuse treatment program and only indicated physical presence at an outpatient clinic or a treatment or supportive facility where abstinence monitoring was regularly performed. By using Swedish registers to assess these variables, it was not possible to control for details regarding the interventions, such as what additional interventions (e.g. pharmacological or psychosocial treatment) were provided. Consequently, we were not able to explore which specific elements of the substance abuse outpatient visits were related to the reduced crime rates observed in the study.

General criminal recidivism was defined as new convictions after inclusion to the study/baseline assessment and was assessed with official recidivism data. Given that new convictions constitute a higher threshold for criminal recidivism, as opposed to new arrests or charges, the crime relapse rates of the study may have been underestimated. In addition, we did not have access to data on potential furlough times. Thus, when calculating time at risk we excluded time-periods during which the participants might have been on furlough. This might have led to an underestimation of time-at risk given that such time periods should have been included in time at risk.

Some strengths of the present study should also be emphasized. As described, the current study was based on an observational study design. Observational studies are open to confounding but we computed a stratified Cox-regression model adjusting for confounders that were constant within each participant during the follow-up period. According to the results of this analysis, the rate of criminal recidivism was reduced by 75% when each person served as his or her own control, reinforcing the result of the extended Cox-model. This suggests that the relationship between participation in outpatient substance abuse interventions and criminal recidivism may not explained by confounders that concern differences between those participating and not participating in the outpatient visits, respectively. Performing this analysis should be considered a methodological strength given that it should reduce the risk of confounding, and in turn increase the internal validity of the study.

Our aim was to include a sample with mental health and substance use problems as well as manifest criminal behavior. All participants had been referred for FPA and a majority (98%) reported at least one prior psychiatric symptom according to the ASI-6. In addition, we used systematic inclusion criteria to screen for hazardous use of alcohol and/or drugs, and the participants had established criminal behavior with a mean number of over five previous convictions according to the ASI-6. Consequently, we believe that we were able to include a sample of offenders with mental health and substance use problems as well as manifest criminal behavior, in line with our intentions. This should be considered a strength of the study.

Furthermore, given that the data on substance abuse intervention participation and general recidivism were collected through Swedish registers, they can be considered as valid (i.e. this information was not based on self-report) [48]. The research assistants collecting the data had received adequate assessment training according to Swedish national standards prior to recruiting the participants. All assessment tools used in the study have demonstrated reliability in earlier research [31,34,36]. Thus, reliability of the data can be assumed.

The long follow-up period of approximately three years should be considered a methodological strength given that earlier studies on offenders with mental health problems and substance use problems have had shorter follow-up periods [5,19]. Finally, as far as we know, only two studies have explored the relationship between participation in substance abuse interventions and criminal recidivism in similar populations in a community setting [6,19]. Our findings add knowledge to this topic.

## Conclusions

In a Swedish sample of offenders with mental health and substance use problems, participation in planned visits to specialized substance abuse outpatient clinics was associated with substantially lower crime relapse rates. Participation in substance abuse outpatient interventions could be regarded as an important part of a clinical risk management strategy in the community. Treatment providers need to motivate offenders to start and remain in contact with specialized substance abuse outpatient clinics.

Future studies on this topic should explore which specific elements of substance abuse interventions might be beneficial. Also, future research should study other outcomes of substance abuse outpatient participation such as mental health and substance use problems in order to further explore the efficiency of this intervention. When possible, research on these topics should include appropriate control groups and randomization to settings in order to draw conclusions about causality.
